# Assessment of ocular manifestations in spondyloarthropathies: An observational study

**DOI:** 10.6026/973206300214207

**Published:** 2025-11-15

**Authors:** Yasha Bandil, Dhirendra Kumar Pandey, Aashi Jain, Praher Shrivastava, Dileep Dandotiya

**Affiliations:** 1 Department of Ophthalmology, MGM Eye Institute, Raipur, Chhattisgarh, India; 2 Department of Ophthalmology, Birsa Munda Government Medical College, Shahdol, Madhya Pradesh, India; 3 Department of Ophthalmology, Shyam Shah Medical College and Associated SGMH Hospital, Rewa, Madhya Pradesh, India; 4 Department of Ophthalmology, Atal Bihari Vajpayee Government Medical College, Vidisha, Madhya Pradesh, India; 5 Department of Community Medicine, Chhindwara Institute of Medical Sciences, Chhindwara, Madhya Pradesh, India

**Keywords:** Spondyloarthropathies, ankylosing spondylitis, uveitis, ocular manifestation

## Abstract

Spondyloarthropathies (SpA) are systemic inflammatory disorders often presenting with ocular involvement. This observational study conducted at the Department of Ophthalmology, SSMC Rewa, evaluated ocular manifestations among patients diagnosed with various spondyloarthropathies. Ankylosing spondylitis was the most frequent subtype associated with ocular involvement (49%), with uveitis (25%) and dry eye (9%) being the predominant findings. Ocular manifestations were more common in males aged 30-39 years and showed a significant association with longer disease duration. Anterior uveitis emerged as the most prevalent manifestation, highlighting the importance of early detection and management to prevent vision-threatening complications.

## Background:

With a prevalence of 1.5% to 2% in the general population, spondyloarthritis (SpA) is a category of rheumatic disorders that are somewhat prevalent [1]. Ankylosing spondylitis (AS), psoriatic arthritis (PsA), reactive arthritis (ReA), arthritis of inflammatory bowel disease (AIBD), juvenile spondylitis (jSpA) and undifferentiated spondyloarthritis (uSpA) are the five conditions that make up spondyloarthritis (SpA). The pathophysiological underpinnings and clinical patterns of many illnesses are similar [2]. A number of extra-articular symptoms, including uveitis, enthesitis, skin lesions, apical lung fibrosis, valvular aortic insufficiency and cardiac blockages, may accompany spinal and peripheral joint involvement [3]. Ocular manifestations were shown to be the most prevalent extra-articular manifestation. SpAs and other autoimmune diseases have harmful systemic and ocular repercussions. SpA is characterized by peripheral symptoms, including inflammation of the fingers and toes (dactylitis) and entheses (enthesitis). SpA is commonly associated with ocular issues, with uveitis being the most common, occurring in over 40% of patients. Both viral and non-infectious uveitis can occur as a local process or as a component of a systemic disease, such as rheumatology. According to the literature, people with rheumatological disease-associated uveitis have a greater recurrence rate, bilateral involvement and more posterior synechiae than those without [4]. Based on factors like laterality, clinical course and anatomical location, uveitis can be categorized. Hence, uveitis can be classified as follows based on its anatomical location: anterior uveitis, which involves inflammation of the iris or ciliary body (also known as iridocyclitis or iridocyclitis); posterior uveitis, which involves inflammation of the choroid or, as a result, the retina (also known as uveitis or chorioretinitis); intermediate uveitis, which involves inflammation that is restricted to the vitreous, peripheral retina and ciliary body pars plana (also known as pars planitis); or panuveitis, which involves inflammation of the entire uvea, including the iris, ciliary body and choroid. The precise etiology of uveitis and SpA is yet unknown. The two illnesses, however, seem to be closely related, resulting from the interplay of a particular, typically prevalent genetic inheritance, extrinsic elements like the microbiome, bacterial infections, or mechanical stress, as well as immune system activation and inflammation [5]. When an episode of uveitis occurs suddenly and lasts less than three months, it is referred to as acute. Recurrent episodes are defined as those that occur more than three months apart and are accompanied by periods of no disease activity without treatment. Furthermore, uveitis that returns within three months of stopping treatment is referred to as persistent. The illness is deemed inactive when the anterior chamber is cell-free [6]. Each person has a different presentation of ocular manifestation, which can range from mild to severe. If left untreated, uveitis raises the possibility of blindness from other chronic ocular inflammatory consequences. An accurate diagnosis and timely treatment by an ophthalmologist working with a rheumatologist may help to lessen the upsetting eye complications and sequelae. The type of ocular involvement in childhood and adult SpA was shown to be similar in a meta-analysis conducted by Turk *et al.* In contrast to PsA that develops in children, uveitis was less common in adult-onset PsA [7]. Therefore, it is of interest to describe the spectrum and frequency of ocular manifestations among patients with different types of spondyloarthropathies.

## Aims of the Study: 

The purpose of this study was to evaluate the different ocular symptoms of different spondyloarthropathies in a tertiary care hospital.

## Materials and Methods:

This cross-sectional, observational, Hospital-based study was conducted in the Department of ophthalmology, SGMH, Rewa, India, from January 2021 to September 2022 (21 months). All Patients attending the outpatient clinics with a definitive diagnosis of Spondyloarthropathies were enrolled in this study.

## Inclusion criteria:

[1] Patients ≥18 years of age

[2] All the diagnosed cases of various spondyloarthropathies

[3] Patients who provided written informed consent for the study

## Exclusion criteria:

[1] Children less than 18 years of age

[2] Patients with previous ocular trauma, surgery, or laser procedure in the eye

[3] Patient with any other known uveitic entities like Fuchs heterochromic iridocyclitis, Posner-Schlossman syndrome,

[4] Patients with any other ocular or systemic disease (other than SpA) which may be associated with or masquerade as uveitis were excluded from the study.

All patients had a detailed history and ophthalmological evaluation to look for ocular manifestations of SpA. They were examined using a slit lamp biomicroscope, ophthalmoscope and tear film tests.

## The following was noted in the patients-

[1] Relevant ocular history, history of pain, redness and defective eye vision and duration.

[2] Duration of Spondyloarthropathy

[3] Best corrected visual acuity

[4] Slitlamp evaluation of the anterior segment.

[5] Posterior segment evaluation with an indirect ophthalmoscope and

[6] Slitlamp biomicroscopy using 90D.

[7] IOP measurement using a Non-contact tonometer.

[8] Dry eye evaluation by Schirmer test using whatmann 41 filter paper. Schirmers 1 tests both basal and reflex tear secretion.

[9] Schirmers 2 tests the basal secretion. It is done after applying paracaine drops.

[10] Tear break-up time- a TBUT of <10 seconds was taken as abnormal.

## Ethical consideration:

This study was approved from the Institutional Review Board.

## Statistical analysis:

Analysis of data was performed using SPSS Version 20.0. Continuous parametric variables were expressed as means and standard deviation; Categorical variables were expressed as percentages. Comparison of categorical variables between two groups was done using Chi square test. P-value of less than 0.05 was considered statistically significant.

## Results:

It was observed that almost half of the study group had ankylosing spondyloarthropathy, followed by 17 cases had inflammatory bowel associated arthropathy, 15 had undifferentiated spondyloarthropathies, 13 cases had psoriatic, while 6 cases had reactive arthritis (Figure 1 - see PDF). IBDAA and PsA were significantly associated with age of the patient. PsA was observed to be significantly higher amongst > 30 years, while IBDAA was observed amongst < 30 years patient. There was no statistically significant association of gender with type of spondyloarthropathy. Ankylosing spondylitis was significantly higher amongst cases residing in urban area. In contrast, IBDAA was observed to be significantly associated with cases residing in rural areas. Ankylosing spondylitis was significantly present in cases with disease duration of > 5 years. Pain, redness and Ciliary congestion were significantly associated with ankylosing spondylitis. Patients with Psoriatic arthroplasty were also found to have a significant association with foreign body sensation and diminution of vision. Reactive arthritis was significantly associated with blanching observed with 2.5% Phenylephrine. It was observed that pupillary reaction, corneal deposits, scleral congestion and presence of Synechiae was not significantly associated with any spondyloarthropathy. Ankylosing spondylitis had a significantly higher proportion of developing uveitis compared to any other spondyloarthropathy (Figure 2 - see PDF). We have observed that dry eye, anterior chamber reaction and lens involvement was not significantly associated with any spondyloarthropathy (p>0.05) (Tables 1-3 - see PDF).

## Discussion:

Among the seronegative spondyloarthropathies, uveitis in ankylosing spondylitis is the most common ocular manifestation. The various manifestations include uveitis, the most common finding, followed by dry eye, episcleritis and scleritis. Among them, uveitis is a potentially vision-disabling complication and needs to be detected early and treated promptly to avoid irreversible vision loss. We have found that the majority of the cases had ankylosing spondyloarthropathy, followed by inflammatory bowel associated arthroplasty and undifferentiated spondyloarthropathies, similar findings were observed by Zeboulon *et al.* [8] and Rosenbaum *et al.* [9]. There was a male preponderance in the current study but this is probably due to more access of male patients to health care services in our country. According to Monnet *et al.* [10] observed that male-to-female ratio was 1.3 to 1. In the present study IBDAA and PsA were significantly associated with age of the patient. PsA was observed to be significantly higher amongst > 30 years, while IBDAA was observed amongst < 30 years patient; our results were comparable with the Khan *et al.* [11]. We have found that Pain, redness and ciliary congestion were significantly associated with ankylosing spondylitis, in agreement with the Pandey *et al.* [12] and Ninan *et al.* [13]. The prevalence of uveitis increases with disease duration, most of the study subjects had duration of the disease was 5-10 years, consistent results reported by Zeboulon *et al.* [8] and Parida *et al.* [14]. There is an increased incidence of dry eye in patients with seronegative spondyloarthropathies. In our study there was reduced TBUT and Schirmers level in the patients. Similar results were reported by Chauhan *et al.* [15]. In the current study there was a strong correlation between the occurrences of anterior uveitis in Ankylosing spondylitis. Psoriatic arthroplasty were also found to have a significant association with foreign body sensation and diminution of vision in this research, concordance with the Cantini *et al.* [16]. Our findings are in alignment with the recently published cross-sectional study [17] which likewise reported that anterior uveitis was the most common ocular manifestation (19 %) among seronegative spondyloarthropathies and emphasised the imperative of early recognition and treatment to forestall irreversible vision loss.

## Limitations:

There few limitation of this study was less sample size and no facility to conduct HLA B27 test.

## Conclusion:

Data show that common spondyloarthropathy causing the ocular manifestation is found to be ankylosing spondylitis. Most common ocular manifestation found was uveitis, dry eye, episcleritis and scleritis. Uveitis may lead to defective vision and many other serious complications; hence a high degree of suspicion is required for early diagnosis and prompt treatment. Due to multi system involvement a combined treatment approach involving screening and early treatment of ocular manifestations in spondyloarthropathies patients is required to reduce the ocular morbidity of the patient and for complete patient care.

## Source of funding:

None
